# Novel homozygous nonsense mutation associated with Bardet–Biedl syndrome in fetuses with congenital renal malformation

**DOI:** 10.1097/MD.0000000000030003

**Published:** 2022-08-12

**Authors:** Meiying Cai, Min Lin, Na Lin, Liangpu Xu, Hailong Huang

**Affiliations:** a Medical Genetic Diagnosis and Therapy Center, Fujian Maternity and Child Health Hospital College of Clinical Medicine for Obstetrics and Gynecology and Pediatrics, Fujian Medical University, Fujian Key Laboratory for Prenatal Diagnosis and Birth Defect, Fuzhou, China.

**Keywords:** Bardet–Biedl syndrome, congenital renal malformation, rare autosomal recessive genetic disorder, whole exome sequencing

## Abstract

**Background:**

The Bardet–Biedl syndrome (BBS) is a rare autosomal recessive disorder, characterized by clinical and genetic heterogeneity. BBS is more commonly reported in adults and children than in fetuses. Here, a retrospective study on 210 fetuses with congenital renal malformation was conducted.

**Methods:**

The fetuses were diagnosed using invasive prenatal tests, including chromosome karyotype analysis, whole exome sequencing (WES), and single-nucleotide polymorphism array. We found the intrauterine phenotype of a fetus presenting enlarged kidneys, enhanced echo, and oligohydramnios; therefore, the fetus was characterized to have BBS.

**Results:**

Chromosome karyotype analysis presented normal results. Analysis using an Affymetrix CytoScan 750K array revealed 2 homozygous regions. However, WES revealed a homozygous mutation of c.1177C>T (p.Arg393*) on exon 12 of *BBS1* and a heterozygous variation of c.2704G>A (p.Asp902Asn) on exon 22 of *CC2D2A*. The American College of Medical Genetics and Genomics guidelines identified c.1177C>T and c.2704G>A as a pathogenic mutation and of uncertain significance, respectively. Sanger sequencing identified heterozygous mutation, that is, c.1177C>T and heterozygous variation, that is, c.2704G>A in the parents of the fetus.

**Conclusions:**

WES identified a novel homozygous nonsense mutation c.1177C>T in *BBS1* of a Chinese fetus with congenital renal malformation. This finding provides insight into the *BBS1* mutations in Asian populations in general and shows the necessity of genetic counseling.

## 1. Introduction

Bardet–Biedl syndrome (BBS; MIM 209900) is a rare autosomal recessive disorder. The prevalence of BBS in the European and North American populations is very low, that is, approximately 1/160,000 to 1/140,000,^[1]^ and that in Asian populations is even lower, that is, approximately 1 in 18 million.^[2]^ BBS is characterized by intellectual disability, retinopathy pigmentosa, polydactyly (toes), obesity, gonadal hypoplasia, renal dysplasia, and short stature.^[3,4]^ Secondary clinical manifestations include developmental disability, motor and neurological dysfunction, speech disorders, and behavioral abnormalities, as well as eye cataracts, strabismus, and astigmatism.

A total of 21 genes associated with BBS phenotypes have been identified so far,^[5,6]^ and different BBS-related genes result in different morbidities. For example, BBS related to *BBS1*,^[7]^ *BBS2*,^[8]^ *BBS6*,^[9]^ *BBS9*,^[10]^ *BBS10*,^[11]^ and *BBS12*^[12]^ mutations accounted for 23.3%, 8.1%, 5.8%, 6.0%, 20%, and 5% of the cases, respectively.^[13]^ The mutation frequencies of the BBS genes differ among ethnic groups. Mutation frequency of *BBS1* is high in European populations, thereby leading to the occurrence of BBS, while *BBS7* mutation is more commonly found in the Chinese population. Although mutations in 21 BBS genes that can result in the BBS phenotypes have been identified, only 80% of the patients show mutations located in these genes, and the remaining 20% of BBS instances are unrelated to these genes. Therefore, further identification of other BBS-related genes is necessary. Several challenges still exist regarding the genetic diagnosis and treatment of this disease.

BBS is a relatively rare condition and has a very high tendency of causing disabilities as it heavily damages multiple systems and organs. At present, our understanding of the pathogenic molecular mechanism of BBS is incomplete, and no special treatments targeting this condition have been designed.^[14]^ Therefore, avoiding consanguineous marriage and using effective prenatal screening are important preventive measures to lower the occurrence of BBS.^[15,16]^

To our knowledge, no instances of BBS associated with the *BBS1* variants have been reported in the Chinese population. We retrospectively analyzed 210 fetuses with congenital renal malformation, and among these we diagnosed 1 fetus with BBS1 mutation in the Chinese population. We further analyzed their pedigrees to explore the relationship between intrauterine phenotypes and fetal genotypes to improve the diagnostic and monitoring methods, as well as our understanding of the disease.

## 2. Methods

### 2.1. Ethical approval and consent to participate

The studies were approved by the ethics committee at the Fujian Provincial Maternal and Child Health Hospital (no. 2014042). All patients signed written-informed consents to participate in this study.

### 2.2. Study participants

A retrospective study on 210 fetuses with congenital renal malformation in the Fujian Provincial Maternal and Child Health Hospital was conducted from November 2016 to February 2021. These fetuses were diagnosed using invasive prenatal tests. Amniocentesis, chorion villus sampling, or blood sampling from the umbilical cord was performed according to the pregnant woman’s gestational stage.

### 2.3. Chromosome karyotype analysis

Transabdominal amniocentesis was performed using ultrasound, and 40 mL of amniotic fluid was extracted. Of the extracted amniotic fluid, 20 mL was cultured in vitro under aseptic conditions, and the remaining 20 mL was used for DNA extraction. The cultured cells from the amniotic fluid were harvested, fixed, and prepared for karyotyping and G banding. Chromosomal abnormalities were described according to the International System of Human Cytogenetics Nomenclature (2016). Forty karyotypes were counted in each case, and 5 were analyzed karyotypes; the count and analysis of karyotypes were increased in case of any abnormality.

### 2.4. Single-nucleotide polymorphism array

Experiments were conducted in strict accordance with the standard operating procedures provided by Affymetrix. The data were analyzed using CHAS 2.0 software. The single-nucleotide polymorphism (SNP) array structure was analyzed in combination with the relevant databases to determine the nature of the obtained copy number variation (CNV). The reference databases included DGV (http://dgv.tcag.ca/dgv/app/home), DECIPHER (http://decipher.sanger.ac.uk/), OMIM (http://www.omim.org), ISCA (http://www.iscaconsortium.org), and CAGdb (http://www.cagdb.org/). CNVs can be divided into 5 categories,^[17,18]^ that is, pathogenic, possibly pathogenic, of uncertain clinical significance (VUS), possibly benign, and benign. For the VUS category, it is recommended to conduct SNP analysis in the fetal cells isolated from maternal peripheral blood in combination with pedigree analysis to further clarify the nature of CNV.

### 2.5. Whole exome sequencing

A library was prepared from the fetal DNA. Then, the exons of the target genes and DNA in the adjacent shear region were captured and enriched using a Roche KAPA HyperExome chip. Finally, mutations were detected using the MGISEQ-2000 sequencing platform. The quality control index of sequencing data was as follows: the average sequencing depth of the target region was ≥180×, and loci with average depths >20× in the target region accounted for over 95% of the total loci. Sequenced fragments were compared with the UCSC hg19 human reference genome to remove duplicates. INDEL and genotype detection were performed using GATK. ExomeDepth was used to detect CNV at the exon level, and genes were named according to the Human Genome Organization Gene Nomenclature Committee (HGNC). Variants were named according to Human Genome Variation Society (HGVS) nomenclature. The following reference databases and prediction software versions were used: Clinvar (2020-03-16), ESP6500 (V2), 1000 Genomes (phase 3), GnomAD (r2.0.1), ExAC (r0.3.1), BPGD* (V3.1), SecondaryFinding_Var*(v1.1_202.3), dbscSNV (1.1), SpliceAI (1.3), dbNSFP (2.9.1), SIft, MutationTaste, and Polyphen2. The pathogenic properties of the variants were classified in accordance with the sequence variation interpretation guidelines recommended by the American Society of Medical Genetics and Genomics (ACMG) and the American Society of Molecular Pathology.^[19–22]^ The Clingen Working Group on the Interpretation of Sequence Variations and the Society for Clinical Genome Sciences were consulted to refine our interpretation of the guidelines.

### 2.6. Sanger sequencing to validate pedigree analysis

Peripheral blood samples (5 mL) from both parents of the fetus were collected, and ethylene diamine tetraacetic acid was used to prevent coagulation. DNA was extracted using a DNA extraction kit (Tiangen Biochemical Technology Co., Ltd, Beijing, China) according to the manufacturer’s instructions. Suspected pathogenic loci found by whole exome sequencing (WES) were amplified using polymerase chain reaction. After purification and quantification, the products were sequenced using an ABI 3130 Genetic Analyzer, and the obtained sequences were compared with human wild-type sequences.

## 3. Results

### 3.1. Clinical phenotype

**Figure 1.**
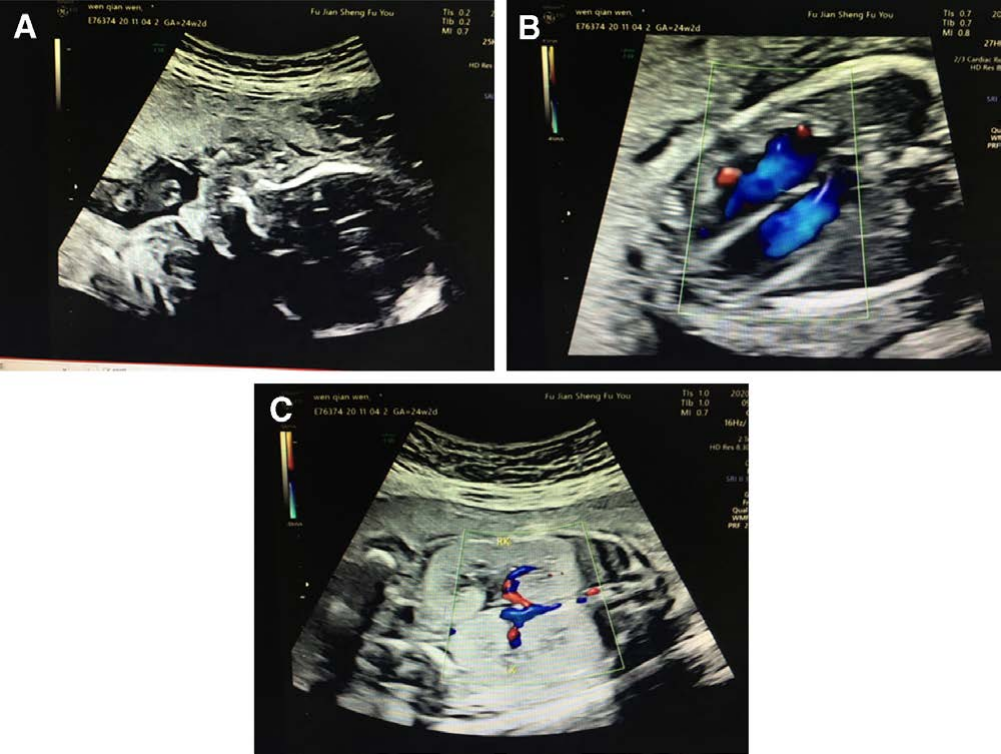
Intrauterine ultrasound phenotype of the fetus. (A) Ultrasound of the fetus at 24 ^+2^ gestational week; single pregnancy in utero. (B) Ultrasound of the fetus at 24 ^+2^ gestational week; both kidneys were enlarged, the echo was enhanced, and infantile polycystic kidney was suspected. (C) Ultrasound of the fetus at 24 ^+2^ gestational week; the amniotic fluid index was 2.9 cm, showing a slightly sparse state.

Among the 210 fetuses with congenital renal malformation, the intrauterine analysis of 1 fetus exhibited enlarged kidneys and enhanced echo; this resulted in the diagnosis of suspected to be infantile polycystic kidney disease. The amniotic fluid index was slightly low (2.9 cm; Fig. 1A–C).

### 3.2. Chromosome karyotype analysis

**Figure 2.**
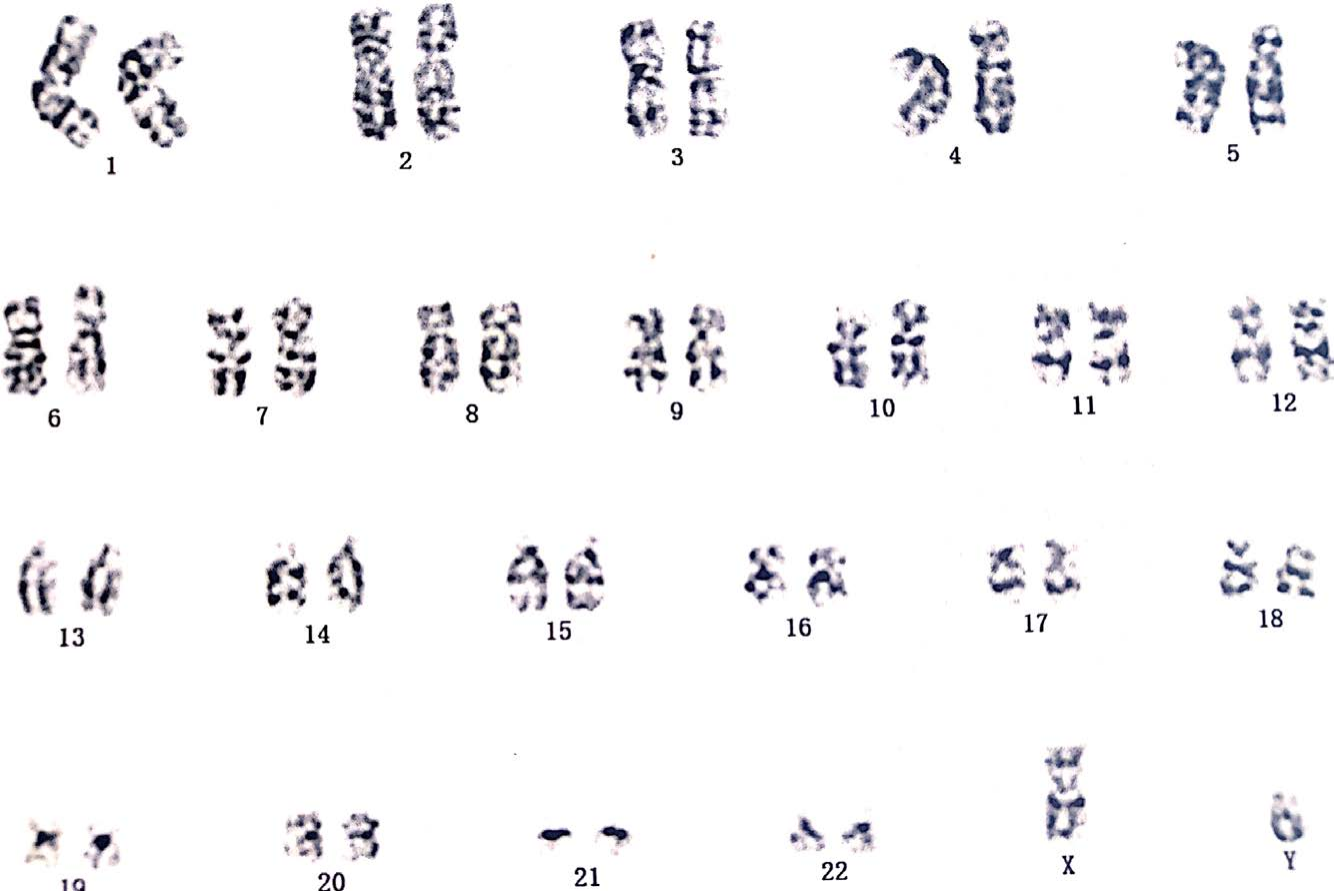
Karyotype of the fetus from amniotic fluid.

Prenatal cytogenetic analysis of amniotic fluid revealed a normal karyotype: 46, XY (Fig. 2).

### 3.3. SNP array

**Figure 3.**
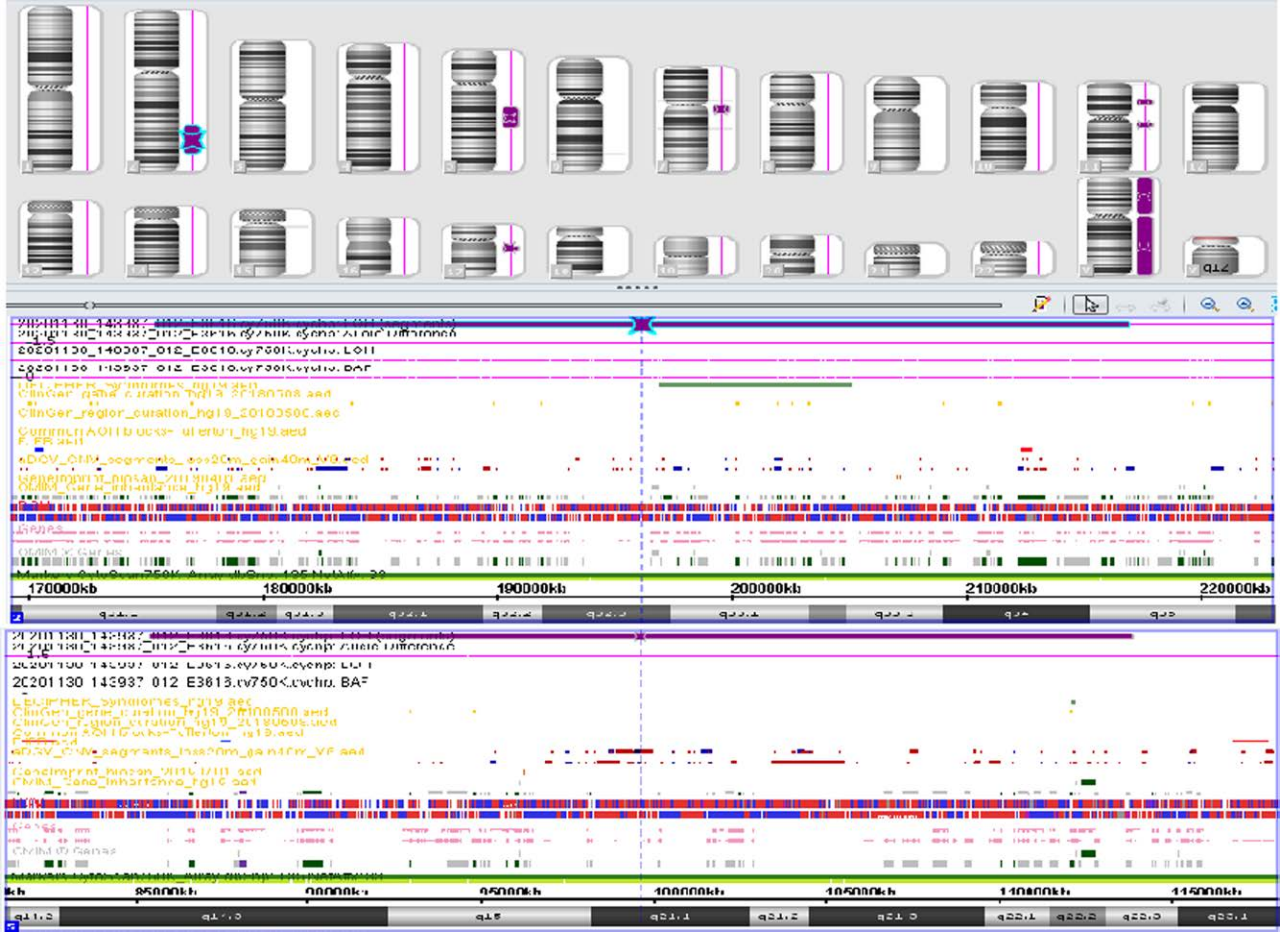
SNP analysis showing 2 homozygous regions.

SNP analysis indicated a homozygous region of 41 Mb in the q31.1q35 region of chromosome 2 containing 153 OMIM genes and a homozygous region of 28 Mb in the q14.3q22.3 region of chromosome 5 containing 51 OMIM genes. No imprinted genes in these regions were identified; however, an increased risk of recessive genetic diseases caused by homozygous mutations was indicated (Fig. 3).

### 3.4. WES

**Figure 4.**
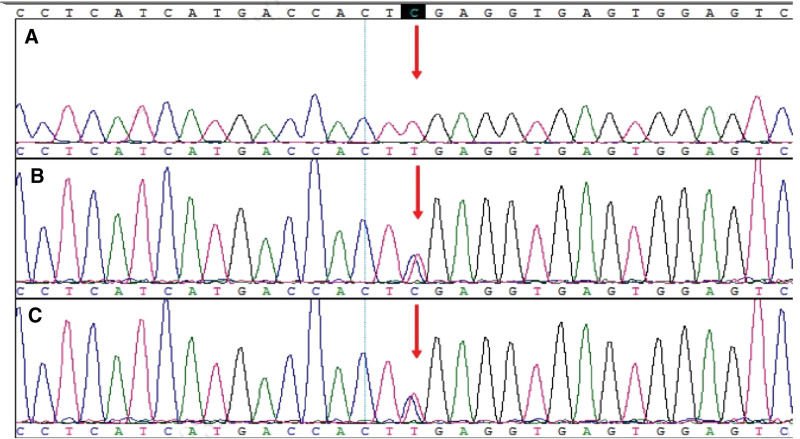
*BBS1* sequencing of the fetus and parents. (A) homozygous variation of c.1177C>T (NM_024649.4, p.Arg393*) in exon 12 of *BBS1* in fetus; (B) heterozygous variation of c.1177C>T (NM_024649.4, p.Arg393*) in exon 12 of *BBS1* in the mother; and (C) heterozygous variation of c.1177C>T (NM_024649.4, p.Arg393*) in exon 12 of *BBS1* in the father.

WES revealed homozygous variation of c.1177C>T (NM_024649.4, p.Arg393*) in exon 12 of *BBS1* in the fetus (Fig. 4). This variation leads to the premature termination of protein synthesis at amino acid position 393, resulting in the production of truncated proteins. This ultimately affects protein function. In accordance with the ACMG guidelines, c.1177C>T was identified as a pathogenic mutation, with the PVS1, PM2, and PM3 criteria.

These criteria are defined by several standards. PVS1 occurs when the pathogenic mechanism underlying disease is a loss of function mutation. This mutation can occur as a nonsense, frameshift, or start codon mutation, depending on the deletion of 1 or more exons. PM2 occurs when variations are not found in control population in the ESP, 1000 genome, and EXAC databases. PM3 occurs when recessive genetic diseases and pathogenic variants are detected at the trans position.

**Figure 5.**
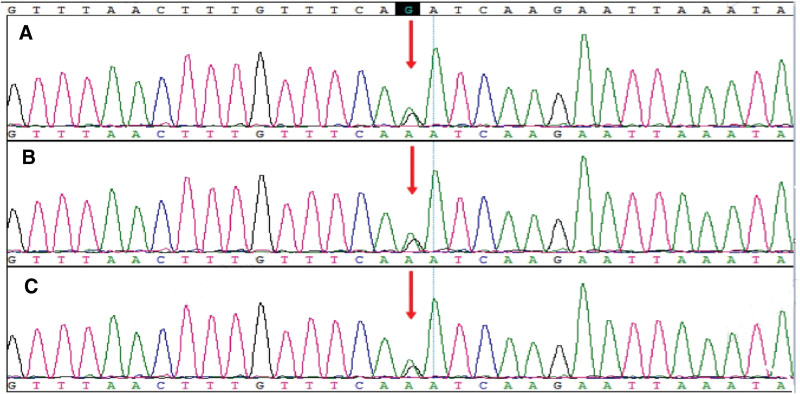
*CC2D2A* sequencing of the fetus and parents. (A) heterozygous variation of c.2704G>A (NM_00108052.2.2, p.Asp902Asn) in exon 22 of *CC2D2A* in the fetus; (B) heterozygous variation of c.2704G>A (NM_00108052.2.2, p.Asp902Asn) in exon 22 of *CC2D2A* in the mother; and (C) heterozygous variation of c.2704G>A (NM_00108052.2.2, p.Asp902Asn) in exon 22 of *CC2D2A* in the father.

WES further revealed a heterozygous variation, that is, c.2704G>A (NM_00108052.2.2, p.Asp902Asn) in exon 22 of *CC2D2A* of the fetus (Fig. 5). According to the ACMG guidelines, c.2704G>A is a variant of unknown significance (PM2).

### 3.5. Sanger sequencing for validation of pedigree

Sanger sequencing identified heterozygous mutations at the same gene positions in the DNA samples of the parents. *BBS1* of the parents exhibited heterozygous variation of exon 12 c.1177C>T (NM_024649.4, p.Arg393*) (Fig. 3). The parents also displayed heterozygous variation of *CC2D2A* on exon 22 c.2704G>A (NM_00108052.2.2, p.Asp902Asn) (Fig. 4).

### 3.6. Pregnancy outcome

The pregnancy was terminated at 25 weeks of gestation, and the parents of the fetus did not provide consent for a postinduction autopsy.

## 4. Discussion

In this study, an intrauterine ultrasound was conducted to determine the phenotype of a fetus with bilateral-kidney enlargement, enhanced echo, polycystic kidney, and an amniotic fluid index of 2.9 cm (a low level at 24 ^+ 2^ weeks of gestation). We first conducted traditional karyotyping and SNP analysis for genetic testing of the fetus. Karyotyping showed no abnormalities. SNP analysis showed no imprinted genes in the 2 homozygous regions, but revealed an increased risk of recessive genetic disease caused by homozygous mutation. WES revealed homozygous variation of c.1177C>T (NM_024649.4, p.Arg393*) in exon 12 of *BBS1*. Sanger sequencing identified heterozygous mutations in the same positions of genes in the parents of the fetus. These data are consistent with an autosomal recessive inheritance of BBS.

*BBS1* (OMIM:209901) is located on chromosome 11q13 and is also known as *BBS2L2*. Presently, 94 pathogenic variants of *BBS1* have been reported by Human Gene Mutation Database. *BBS1* mutation is the most common cause of BBS and is responsible for 25% of all BBS incidences. The type of mutation varies among ethnic groups, with the most common *BBS1* variant (p.M390R) accounting for approximately 80% of all *BBS1* mutations in the European population.^[23,24]^ Mykytyn et al^[25]^ conducted genetic screening on 129 patients with BBS and found that 30% of these patients possessed at least 1 *M390R* mutation. BBS proteins encoded by different BBS genes are functional throughout the formation of the BBS complex, including BBSome, which consists of 7 BBS proteins (BBS1, BBS2, BBS4, BBS5, BBS7, BBS8, and BBS9).^[26–29]^ *BBS1* mutation results in abnormal function of the BBSome, which in turn affects the function of microcilia and other systems in the body.^[30]^ The homozygous variation of c.1177C>T (NM_024649.4, p.Arg393*) in exon 12 of *BBS1* has not been reported in the Chinese population.

Most *BBS1* variants include missense, deletion/insertion, and splicing mutations and produce typical BBS phenotypes.^[31–34]^ Recent studies indicate that 90% of the BBS patients exhibit retinal degeneration,^[35]^ 90% have abnormal renal development and function,^[36]^ and 72% to 92% are obese.^[37]^ Additionally, 63% to 81% of the patients have polydactyly/deformity,^[38]^ and more than half of the patients exhibit intellectual disability and/or gonadal dysplasia.^[39]^ The fetus in this study exhibited a nonsense *BBS1* variant with biallelic loss of function mutation. Renal abnormalities in the sonography results of the fetus are consistent with previously reported clinical abnormalities in the renal development in patients with *BBS1* mutations. The parents of the fetus did not provide consent for postinduction autopsy, therefore, whether the fetus had other clinical manifestations associated with *BBS1* mutations could not be explored.

WES can rapidly and efficiently detect all potentially pathogenic mutations at once.^[40]^ However, the associated huge data output poses a great challenge for bioinformatic analysis and clinical interpretation.^[41]^ In this study, WES revealed a heterozygous variation, that is, c.2704G>A (NM_00108052.2.2, p.Asp902Asn) in exon 22 of *CC2D2A* in the fetus. This gene is primarily involved in the development of the COACH syndrome (OMIM:216360), Joubert syndrome 9 (OMIM:612285), and Meckel syndrome 6 (OMIM:612284).

The COACH syndrome is an autosomal recessive inherited disorder,^[42]^ which exhibits intellectual disability, ataxia (owing to cerebellar hypoplasia), and liver fibrosis as the typical clinical features. Joubert syndrome is an autosomal recessive inherited disease,^[43]^ which manifests clinically as cerebellar ataxia, ocular movement dysfunction, vermis hypoplasia, and thickening of the upper cerebellar foot. Meckel syndrome, another autosomal recessive inherited disease,^[44]^ is a fatal disorder associated with multiple congenital anomalies and characterized by clinical features, including brain malformation, polycystic kidney malformation, polydactyl deformity, cleft lip and palate, cardiac abnormality, malformation of the central nervous system, liver fibrosis, and bone dysplasia. Heterozygous variation, that is, c.2704G>A (NM_00108052.2.2, p.Asp902Asn) in exon 22 of *CC2D2A* was identified in the parents of the fetus. Further studies are necessary to determine the relationship of this variation with congenital renal dysplasia.

## 5. Conclusion

In conclusion, we identified a novel nonsense variant c.1177C>T (p.Arg393*) in the *BBS1* gene of a Chinese family. To the best of our knowledge, this pathogenic homozygous variant in *BBS1* is the first to be reported in the Chinese population. Importantly, it is necessary to carry out prenatal genetic diagnosis in subsequent pregnancies by the parents of the fetus, as both carry pathological variants of *BBS1*.

## Acknowledgments

We thank all patients for their participation.

## Author contributions

**Conceptualization:** LX and NL.

**Writing – original draft:** MC.

**Writing – review & editing:** HH revised the article.

**Data curation and formal analysis:** ML.
